# Assessing the Accuracy of Popular Commercial Technologies That Measure Resting Heart Rate and Heart Rate Variability

**DOI:** 10.3389/fspor.2021.585870

**Published:** 2021-03-01

**Authors:** Jason D. Stone, Hana K. Ulman, Kaylee Tran, Andrew G. Thompson, Manuel D. Halter, Jad H. Ramadan, Mark Stephenson, Victor S. Finomore, Scott M. Galster, Ali R. Rezai, Joshua A. Hagen

**Affiliations:** ^1^Rockefeller Neuroscience Institute, West Virginia University, Morgantown, WV, United States; ^2^Department of Chemical and Biomedical Engineering, West Virginia University, Morgantown, WV, United States; ^3^College of Arts and Sciences, Boston University, Boston, MA, United States; ^4^National Football League, Detroit Lions, Detroit, MI, United States

**Keywords:** wearables, root mean square of successive differences, heart rate variability, validation, heart rate, electrocardiogram, photoplethysmography

## Abstract

Commercial off-the shelf (COTS) wearable devices continue development at unprecedented rates. An unfortunate consequence of their rapid commercialization is the lack of independent, third-party accuracy verification for reported physiological metrics of interest, such as heart rate (HR) and heart rate variability (HRV). To address these shortcomings, the present study examined the accuracy of seven COTS devices in assessing resting-state HR and root mean square of successive differences (rMSSD). Five healthy young adults generated 148 total trials, each of which compared COTS devices against a validation standard, multi-lead electrocardiogram (mECG). All devices accurately reported mean HR, according to absolute percent error summary statistics, although the highest mean absolute percent error (MAPE) was observed for CameraHRV (17.26%). The next highest MAPE for HR was nearly 15% less (HRV4Training, 2.34%). When measuring rMSSD, MAPE was again the highest for CameraHRV [112.36%, concordance correlation coefficient (CCC): 0.04], while the lowest MAPEs observed were from HRV4Training (4.10%; CCC: 0.98) and OURA (6.84%; CCC: 0.91). Our findings support extant literature that exposes varying degrees of veracity among COTS devices. To thoroughly address questionable claims from manufacturers, elucidate the accuracy of data parameters, and maximize the real-world applicative value of emerging devices, future research must continually evaluate COTS devices.

## Introduction

The proliferating market for consumer off-the-shelf (COTS) wearables – Forbes forecasted the wearable market to evolve into a $27 billion industry by 2022 – has created an opportunity for consumers to systematically monitor their own health on a regular basis (Bunn et al., 2018; Lamkin, 2018). As the wearables market continually becomes more competitive, options for wearables include fashion commodities such as smart watches and rings, as well as clothing textiles offering dual-purposes beyond visual appeal that, in many cases, provide users with a plethora of health-related data (Waugh et al., 2018; Aroganam et al., 2019; Depner et al., 2020). These data present a unique opportunity to the end-user that affords them the ability to garner actionable insights related to their personal health, which may include stress states and recovery, as well as physical, cognitive, and psychomotor performance (De Arriba-Pérez et al., 2016; Cardinale and Varley, 2017; Bunn et al., 2018; Aroganam et al., 2019). Ultimately, end-users may incorporate wearable-derived physiological data to sense, assess, and subsequently augment their own well-being by deploying acute lifestyle alterations (e.g., sleep more, increase exercise training load) that eventually manifest into chronic, sustained enhancements (Galster and Johnson, 2013).

Of the commonly reported physiological metrics among COTS wearables, heart rate (HR) data are most consistently captured as it has a multitude of applications and is easily interpretable for most end-users (Achten and Jeukendrup, 2003). HR values provide a numeric representation of general functionality within the human body, with lower values more indicative of a body at rest (negligible stressors). Increased HR values are reflective of an individual's cardiovascular workload during physical exertion or stress (Borresen and Lambert, 2008), with higher values typically reflecting increased metabolic demand or a decrease in functional efficiency (e.g., suppressed running economy due to fatigue) (Achten and Jeukendrup, 2003). Modulations in HR over time are indicative of stress adaptation, which may be considered positive (e.g., increased fitness yielding decreased HR during exercise) or negative (e.g., poor nutrition and sleep yielding increased HR at rest), and are often used in medical, sports, and health/fitness settings as a metric for characterizing general health status (Hillman et al., 2003; Borresen and Lambert, 2008). Further, HR is a strong indicator for mortality (Mensink and Hoffmeister, 1997; Watanabe et al., 2001) as cardiovascular complications are the leading cause of death in the United States (Kitamura et al., 2020). However, there remains a theoretical disconnect in the application of HR to assess autonomic regulation, which would provide a better understanding of stress-recovery states in an individual (Tarvainen et al., 2014). The harmony between stress and recovery within the human body is kept in check by the autonomic nervous system (ANS), which comprises a dynamic balance between its two components, the parasympathetic and sympathetic nervous systems (Aubert et al., 2012). Fluctuations in ANS elements are often quantified by assessing an individual's heart rate variability (HRV) (Shaffer and Ginsberg, 2017).

HRV, derived from the inter-beat intervals spanning across consecutive heart beats (Shaffer and Ginsberg, 2017), provides a more in-depth reflection of auto-regularity modulation within the human body (Acharya et al., 2006; Markovics et al., 2018). Standard HRV metrics comprise the time and frequency domains, which oscillate in response to an individual's immediate psychophysiological response to events or stressors (Shaffer and Ginsberg, 2017). Of those metrics, the most commonly reported metrics are the standard deviation of NN intervals (SDNN) and root mean square of successive differences (rMSSD), both representative of the changes in HR cycles within the time domain (Shaffer and Ginsberg, 2017). Of note, rMSSD is the primary and most useful resting HRV time domain metric (Buchheit, 2014; Shaffer and Ginsberg, 2017), as previous research identified it as an indicator of parasympathetic response to stress (Mayya et al., 2015). rMSSD permits quantification of parasympathetic modulation (Stanley et al., 2015) and reflects the cognitive processes and stress states (Mayya et al., 2015) via direct vagus nerve innervation (DeGiorgio et al., 2010; Wang and Huang, 2012). Deviations from normative rMSSD are indicative of sudden death in epilepsy (DeGiorgio et al., 2010; Wang and Huang, 2012), atrial fibrillation (Wang and Huang, 2012) and other cardiovascular related complications such as congenital heart disease and respiratory sinus arrhythmia (Massin et al., 1999; Berntson et al., 2005). In high performing populations (i.e., athletes or active duty military), rMSSD provides insightful information to ascertain changes in psychophysiology (Berntson et al., 2005), with increased values reflective of adaptation to training and a higher level of fitness and recovery state (i.e., sufficient psychophysiological coping with external stressors) (Schmitt et al., 2015). However, by utilizing a combinatorial approach and regularly assessing HR and rMSSD, more objective monitoring of stress resiliency is accomplished (Buchheit, 2014). Specifically, an inverse correlation between HR and rMSSD has been identified such that a decrease in rMSSD and subsequent increase in HR is reflective of the body's stressed response (positive adaptation) to a greater magnitude in external stress stimuli (Bhati and Moiz, 2017). Additionally, most commercial devices that measure HRV report rMSSD, so it can be generally considered a common metric across HRV technologies. Due to the aforementioned reasons, rMSSD was chosen as the primary HRV metric in the present study to investigate the accuracy of various COTS wearables.

Historically, HR and HRV related metrics are most accurately obtained by use of a multi-lead electrocardiogram (mECG), which is often found in clinical-based settings. However, the recent COTS wearables industry surge stimulated the expansion of various types of electrocardiogram (ECG) and photoplethysmography (PPG) devices. The signal captured from ECG based devices represents electrical activity of the heart, whereas PPG COTS devices utilize an optical technique to infer heart rate dynamics from the quantification of volumetric changes in distal blood flow (Allen, 2007; Georgiou et al., 2018). The gold standard for evaluating cardiovascular physiology (Drew et al., 2004) is an mECG device comprised of multiple leads that are strategically placed in close proximity to the heart itself, enabling the signal to noise ratio (SNR) to remain minimal and constant (Kher, 2019). Still, mECG is considerably impractical for the general population to perform more routine (e.g., daily, weekly) HRV evaluations in real-world settings (Castaneda et al., 2018). Not only are the costs and equipment sophistication associated with clinical mECG unreasonable for most users, the degree of expertise necessary to collect and analyze mECG data further limits the practical applications (Smulyan, 2019). As previously mentioned, the demand for the monitoring of HR and HRV related metrics has been remedied through the emergence and proliferation of COTS wearable technologies, by providing a more user-friendly, comfortable, and cost-effective strategy for end-users. Notably, PPG devices remain among the most popular COTS technologies (Castaneda et al., 2018; Henriksen et al., 2018; Bent et al., 2020), with an estimated 20.1 million smartwatches sold in 2019 (Statista, 2019). Generally speaking, PPG sensors are located on the periphery of the body, resulting in a temporal delay in the propagation of the peripheral pulse wave to the wearable device (Georgiou et al., 2018). The distal location of the devices are also known to make them more susceptible to motion artifacts (Lee and Zhang, 2003; Bent et al., 2020). HRV indices such as rMSSD are highly dependent on the signal quality and the duration of signal acquisition. Certainly, both of these signal components can be corrupted by motion artifacts or inferior data quality (Lee and Zhang, 2003; Baek and Shin, 2017; Castaneda et al., 2018) and purport the need for validation against a vetted mECG device.

As the market and demand for wearables continues to expand with an estimated 137 million smartwatches sold by 2022 (Bunn et al., 2018; Pevnick et al., 2018), there remains a critical lack of third-party, independent validations. Research investigating COTS device accuracy, specific to HRV, is limited, despite the established knowledge that tracking HRV provides pertinent insights related to the stress-recovery and performance states across individuals (Makivic et al., 2013; Teisala et al., 2014; Flatt and Esco, 2016; Lischke et al., 2018). The lack of direct validation for COTS technologies that utilize this valuable measurement tool creates concern surrounding the accuracy of decisions being made from its current applications. To date, HRV metric validation efforts are limited to examining device accuracy during bouts of physical activity (Hernando et al., 2016; Bunn et al., 2018; Henriksen et al., 2018), which is likely a result of the growing interest in HRV monitoring for stress adaptations as they relate to sport performance and physical health (Jiménez-Morgan and Mora, 2017). Thorough third-party validations are further limited by manufacturers often incorporating proprietary noise cleaning algorithms (De Arriba-Pérez et al., 2016; Henriksen et al., 2018; Markovics et al., 2018; Bent et al., 2020) to allegedly bolster signal quality, as many popular wearable devices are commonly sensitive to motion artifact (Lee and Zhang, 2003; Yousefi et al., 2013; Waugh et al., 2018). These algorithms are rarely disseminated to consumers or tested by independent researchers (De Arriba-Pérez et al., 2016). The result is a black box effect for both parties, leaving everyone, except for the manufacturers, unaware as to how the physiological metrics are processed (Tuovinen and Smeaton, 2019).

Shortcomings in the extant literature pertaining to HRV commercial technologies are concerning as data derived from these sources may be incorporated into daily decision making by clinicians, researchers, practitioners, or consumers. In 2016, IBM estimated that poor data quality cost the United States $3.1 trillion per year (Redman, 2016). As such, it is paramount that the validity of current COTS technologies be assessed against an mECG so that any and all end-users are afforded the opportunity to sufficiently evaluate the wearable device(s) most suited to their needs.

Therefore, the purpose of the present study was to perform resting-state validations of HR and rMSSD, collected via COTS devices, against a commercially available mECG. A secondary objective was to determine whether or not there were differences in accuracy for measuring rMSSD when comparing commercial ECG and PPG devices. Extending the knowledge of real-life utility (with respect to decision-making and personal health) in commercial wearable devices, specifically those that measure resting state HR and HRV, has profound implications for not only the general consumer, but clinicians, practitioners, and researchers alike. As such, individuals may be afforded greater degrees of confidence when implementing such wearables into daily living, general practices, and/or research protocols.

## Methods

All components of the experimental protocol delineated below were approved by the Institutional Review Board of West Virginia University (Protocol Number 1803027033) for human subject's research. Procedures were compliant with the Declaration of Helsinki guidelines. Written consent was obtained from each participant prior to enrollment.

### Subjects

Five healthy adults, comprised of three males (mean ± SD; age: 20.33 ± 2.08 y; height: 181.19 ± 10.57 cm; weight: 77.11 ± 9.07 kg) and two females (age: 19.50 ± 0.71 y; height: 160.02 ± 7.18 cm; weight: 56.70 ± 3.21 kg) were recruited to collectively perform a total of 148 validation trials. Participants were screened by the American College of Sports Medicine Risk Stratification guidelines and were deemed as “low risk” (Medicine, 2017). Provided that physiological measurements derived from the COTS devices within a single person may be dependent, the COTS devices do not know that and thus measure indiscriminately from person-to-person. The devices themselves are machines by nature that function to objectively measure heart rate signals derived from any human body that they come into contact with. As such, the experimental design deployed in this study enabled ideal conditions for COTS devices to perform at their best ability, which is similar logic to previously published validations on consumer wearables (Burns et al., 2010a; Kaewkannate and Kim, 2016; Nelson and Allen, 2019; Nakano et al., 2020).

### Experimental Design

This study was designed to evaluate the measurement accuracy of various COTS devices purporting the quantification of rMSSD and mean HR. Experimental trials comprised resting-state measurements obtained in the same environmental conditions (e.g., room temperature, ambient lighting) from COTS devices with simultaneous mECG collection as a comparative validation standard. A comprehensive list of the devices and software applications utilized in the present study is provided in Table 1.

**Table 1:** Comprehensive device list.

**Device name**	**Platform used**	**Device type**	**Measurement strategy**
*Shimmer3 mECG Unit*	*iMotions* Software	Multi-lead electrocardiography	ECG 5 leads (RA, RL, LA, LL, chest-V1) Pre-gelled adhesive electrodes
*Polar H10*	EliteHRV HRV4Training	Chest-based strap	ECG Multiple contact sensors on strap
*Firstbeat Textile Strap*	Firstbeat	Chest-based strap	ECG Multiple contact sensors on strap
*OURA (2nd Gen.)*	OURA	Finger-based ring	PPG Contact Infrared (900 nm) Transmission
iPhone 8	Camera HRV Elite HRV HRV4Training	Smartphone	PPG Contact LED

*A summary of the mECG validation standard, the commercial off-the-shelf devices as well as all smartphone application pairings that were analyzed in the present study*.

### Commercial Off-The-Shelf Devices and Measuring HRV

The various COTS devices and applications implemented for direct comparisons to mECG (Shimmer, Dublin, Ireland) included three tangible devices and three third-party applications. Shimmer was previously validated against clinical grade mECG and was incorporated as our mECG validation standard device (Burns et al., 2010a,b; Kerdjidj et al., 2016). Of the devices examined, the 2nd generation Oura smart ring (OURA; OURA, Oulu, Finland) was a finger-based ring whereas both the Polar H10 (Polar, Kempele, Finland) and Firstbeat Textile strap (FSTBT; Firstbeat, Jyväskylä, Finland) were commercial chest-based ECG straps (cECG). Data from OURA and FSTBT were obtained via Bluetooth synchronizing to the companies' smartphone application on an iPhone 8 (Apple, California, United States). The Polar H10 strap was connected via Bluetooth to the third-party smartphone applications HRV4Training (HRV4TR/ECG; HRV4Training, Amsterdam, Netherlands) and EliteHRV (ELT/ECG; EliteHRV, North Carolina, United States). Additionally, PPG comparisons were conducted for the following third-party applications: HRV4Training (HRV4TR/PPG), EliteHRV (ELT/PPG), and CameraHRV (CAMHRV; CameraHRV, Amsterdam, Netherlands). An iPhone 8 camera was utilized for HRV4TR/PPG, ELT/PPG, and CAMHRV such that all three were fingertip-based PPG (fPPG) assessments. Refer back to Table 1 for a summary of how COTS devices and the different software applications were implemented.

### Validation Trials

In order to evaluate PPG and cECG COTS devices, a chest-mounted, 5-lead (RA, RL, LA, LL, chest-V1), mECG device (Shimmer) was worn constantly throughout the duration of each experimental trial (Burns et al., 2010a,b; Kerdjidj et al., 2016). This device was formatted to record data at 512 Hz via the software program, iMotions (Version 8.1, Copenhagen, Denmark). Electrode sites were prepared by abrading the skin with an alcohol prep pad. New mECG leads were checked for moist conduction gel, then placed at the customary right arm (RA), left arm (LA), right leg (RL), and left leg (LL) anatomical locations, according to manufacturer guidelines. A single trial consisted of successive altering of the various PPG/cECG devices. To mitigate device signal acquisition interference, no more than one device was employed at each location (e.g., wrist, finger, chest). For example, no more than two fPPG's (right and left index fingers) were simultaneously collecting data, and only one cECG strap was introduced at a time, ensuring no physical interference from the mECG. Order of device appearance as well as limb placements were randomized prior to the first trial. Trial durations were dictated by a COTS device's default recording periods and were either 3 or 5 min in duration. In summary, devices were introduced based on anatomical location required for data collection (wrist vs. finger vs. chest) and duration of single recording periods (3 or 5 min). The Firstbeat Quick Recovery Test (QRT) and CAMHRV smartphone application recordings were the only COTS technologies requiring 3-min trials, whereas all others spanned 5 min. To perform an HRV (rMSSD) assessment with OURA requires the execution of their custom app feature “Moment,” which then reports both the minimum HR and average rMSSD during a 5-min window. However, the reporting of minimum HR rather than average HR prevented direct comparisons of HR for OURA vs. the other COTS devices assessed in this study. Each device also had its own calibration window between when the “Start” button was pressed and when data collection commenced. This calibration window was predetermined before data collection and incorporated into the procedures. Ultimately, the goal was to ensure all data collection periods were synchronized for simultaneous initiation and cessation of recording periods.

Prior to collection, subjects were instructed to remain in an upright, seated position, with their hands rested in their lap. During instances which required the use of a fingertip for fPPG measurements, participants supinated their hands (rested on their thighs) and were instructed to place the camera of an iPhone 8 on their index finger such that no erroneous pressure originating from the finger was being applied to the iPhone camera. Additionally, participants were asked to continue normal, spontaneous breathing and abstain from any verbal or non-verbal communication throughout the duration of data collection. Time of day (HH:MM:SS), which was obtained from the same internal clock on the computer used for the entirety of data collection, was manually recorded precisely at the moment COTS devices were initiated. Manual time stamps were later used to denote the exact corresponding time interval from mECG to which COTS were compared against. Time spent switching between devices, time between sessions, and time of day for data collection were not controlled because, for the sake of assessments, we aimed for a relatively higher variation in diurnal rhythm to better understand the performance of the commercial devices under slightly different conditions. Moreover, controlling for the above variables would likely not influence the results, as both the mECG and test wearables ought to be measuring the same data (HR and rMSSD) over the same periods in time. For instance, should there be inconsistencies in timing as a result of the participant or investigator, the impact of such stalling on varying HR or rMSSD would be measurable by both the mECG and wearable with proportionate accuracy and reliability (if the COTS device is capable). Following each evaluation time sequence, data from the respective COTS devices were immediately exported and stored in version 16 of Microsoft Excel (Microsoft, Washington, United States) for later comparisons to Shimmer.

### Data Analysis

Raw mECG waveform data (from the Shimmer) were analyzed via Kubios HRV Premium (V3.2.0, Kuopio, Finland) to enable time segmenting, such that comparisons were directly aligned (using the manually recorded timestamps mentioned above) with data exports obtained from each of the COTS devices (Tarvainen et al., 2014). For the measurement of rMSSD from the raw Shimmer signal, data processing was executed within Kubios to enable direct (synchronized with COTS devices) comparisons. Since the HRV analyses primarily focused on rMSSD (i.e., autonomic activity), very low frequency trend components were removed by using the Smoothing priors method. The smoothing parameter was set to λ = 400, which corresponds to a cut-off frequency of 0039 Hz (below the low-frequency band). Finally, inter-beat intervals (IBIs) were extracted from the R-R temporal differences throughout each recording session. For each device, rMSSD was calculated as the square root of the mean squared differences between successive RR intervals for the specified recording period. In summary, HR and rMSSD values from COTS devices were obtained from their companion applications whereas the same metrics derived from the Shimmer signal were analyzed in and extracted from Kubios.

### Statistical Analyses

Analysis began with the calculation of absolute percent errors (APE) with respect to mECG for each available device (listed in Table 1) and metric pairing. Evaluations of mean HR were performed on all COTS devices except for OURA, although the smart ring is included in rMSSD comparisons. The formula used for APE follows:

(1) $$\begin{array}{l} \frac{\left|\text{COTS device measurement}-\text{mECG measurement}\right|}{\text{mECG measurement}}*100 \end{array}$$

Next, the Tukey outlier detection rule was employed to identify any extreme outliers in APE values with respect to each device and metric combination (Pan and Tompkins, 1985). According to this rule, an APE observation is regarded as “extreme” if it lies outside the outer fence of the device/metric APE boxplot, which is defined as 3^*^IQR above the respective third quartile, or 3^*^IQR below the respective first quartile (Dawson, 2011). Removal of these values helped to account for errors in procedure, or problems with Kubios. A total of 20 extreme outliers were identified out of 363 total data points (this explains the varying sample sizes in the results to follow).

From here, summary statistics per device/metric pairing were provided, including mean APE (MAPE). Then, ordered boxplots (determined by MAPE) were constructed to help visualize and compare device performance. A second measure of device performance, Lin's concordance correlation coefficient (CCC), was also calculated (Lin, 1989). This is an established analysis for evaluating agreement (Morgan and Aban, 2016) that has been used in other COTS wearables research validating heart rate measurements (Nelson and Allen, 2019) and validating new methods to measure myocardial blood flow (Dunet et al., 2016). The CCC aims to measure the overall strength of agreement between device measurements and their corresponding mECG measurements by comparing their bivariate relationship to the concordance (identity) line. Similar to the traditional Pearson correlation coefficient, the CCC has a range from −1 to +1, with +1 representing perfect agreement.

Neither the CCC nor APE provide any clinically applicable information on whether the device measurements overestimate or underestimate the mECG measurements (with respect to the metric in question). However, this is a topic of interest thus the Bland Altman (BA) limits of agreement method, which provides measures and visualizations of device bias and precision (or lack thereof), was employed (Bland and Altman, 1986). This method is also recommended for use in evaluating agreement (McLaughlin, 2013), with applications in studies comparing PPG pulse rate variability measurements to ECG HRV measurements (Bánhalmi et al., 2018). For each metric and device combination, an individual BA analysis was conducted. The usual BA statistics [bias, lower limit of agreement (LOA), upper LOA] were presented in tabular form, while the plots were arranged in a grid. Additionally, with respect to each device and metric combination, it was of interest to determine whether significant bias was present. This was achieved by conducting a paired difference *t*-test using a hypothesized mean of 0. Since there were multiple devices in comparison, a Bonferroni correction was applied to the resulting *p*-values. From here, cases where *p* < 0.05 were deemed statistically significant.

Statistical analyses were performed in R version 4.0.0 (Team, 2019). Plots were constructed via the *gridExtra* package (Auguie, 2017) as well as the *tidyverse* packages, which were also utilized for data pre-processing (Wickham et al., 2019). The *blandr* and *rstatix* packages were incorporated to facilitate calculations of Bland Altman statistics (Datta, 2017; Kassambara, 2019).

## Results

### Evaluation of COTS Devices in Reporting Heart Rate

**Figure 1:**
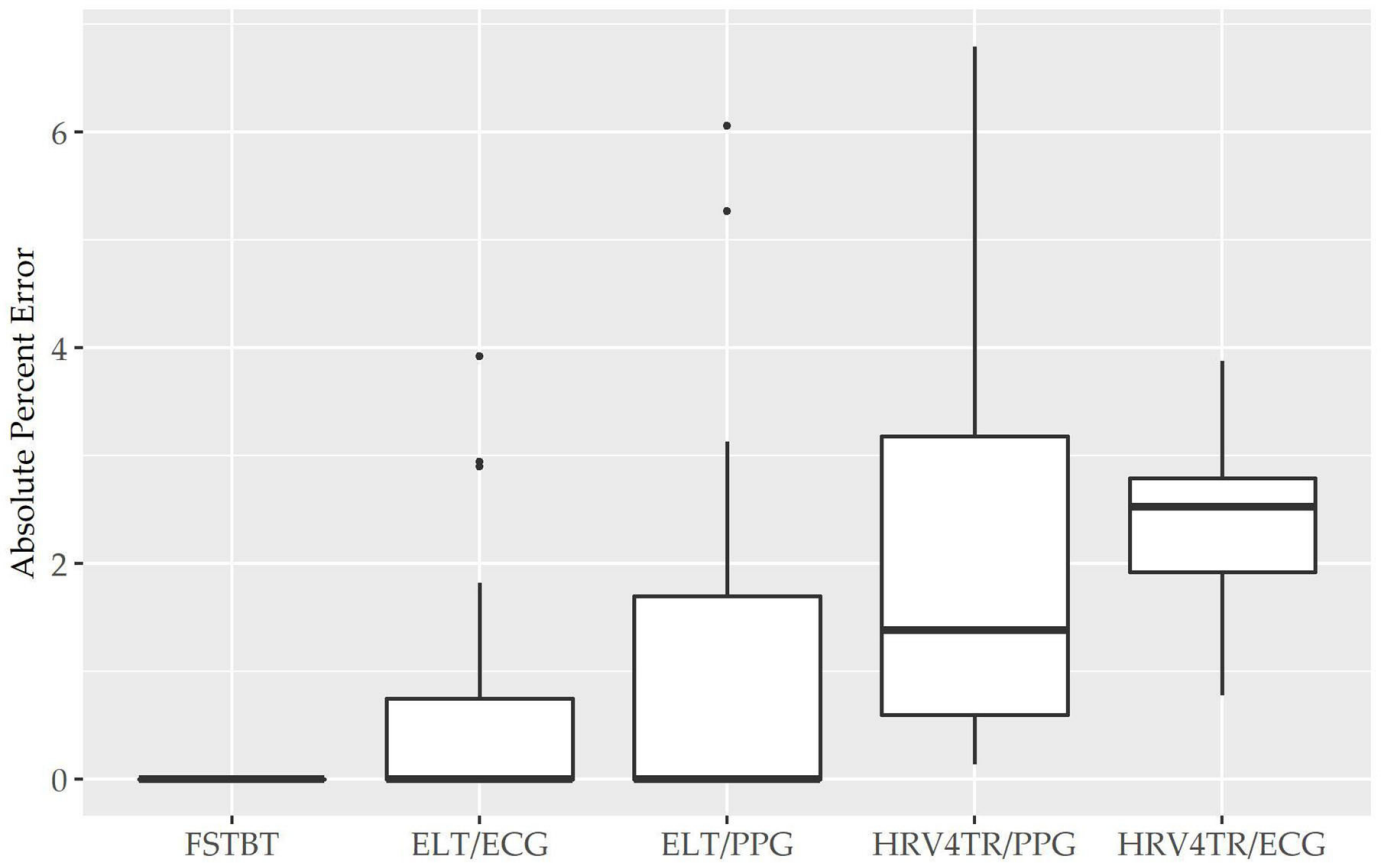
Mean heart rate absolute percent error: box plot visualization of absolute percent error (APE) for the commercial-off-the-shelf (COTS) devices that reported mean heart rate (mHR). COTS devices that are presented include Firstbeat (FSTBT), Elite HRV via electrocardiogram (ELT/ECG), Elite HRV via photoplethysmography (ELT/PPG), HRV4Training via PPG (HRV4TR/PPG), and HRV4Training via ECG (HRV4TR/ECG).

Before determining accuracy in reporting rMSSD, mean HR data from the COTS devices (when available) were first compared to the mECG device via APE. COTS devices were sorted from lowest to highest MAPE values (FSTBT: 0%; CAMHRV: 17.26%, respectively) and are presented in Figure 1. Accompanying summary statistics for absolute percent error appear in Table 2. When measuring HR, the top two performers were FSTBT and ELT/ECG, with reported MAPE values of 0 and 0.69%, respectively. MAPE values were <5% for five of the six COTS devices analyzed. CAMHRV, an fPPG third-party app, reported as the worst device for measuring HR with a MAPE of 17.26%, which was further corroborated by having the highest median (12.03%), maximum (56.03%), and interquartile range (IQR; 15.31%) in comparison to all other COTS devices. For this reason, CAMHRV was omitted from Figure 1 to avoid drastically skewing the scaling of the figure. Otherwise, FSTBT (MAPE; 0%), ELT/ECG (0.69%), ELT/PPG (1.22%), HRV4TR/PPG (2.07%), HRV4TR/ECG (2.34%) were all deemed sufficient at measuring and reporting HR according to APE summary statistics (Table 2).

**Table 2:** Absolute percent error executive summary statistics: mean heart rate.

**Metric**	**Device**	***n***	**MAPE (%)**	**Min. (%)**	**Median (%)**	**Max. (%)**	**IQR (%)**
HR	FSTBT	21	0	0	0	0	0
	ELT/ECG	19	0.69	0	0	3.92	0.75
	ELT/PPG	21	1.22	0	0	6.06	1.70
	HRV4TR/PPG	20	2.07	0.14	1.38	6.80	2.59
	HRV4TR/ECG	21	2.34	0.77	2.53	3.88	0.88
	CAMHRV	23	17.26	0	12.03	56.03	15.31

*Absolute percent error summary statistics are reported for all devices that provided mean heart rate measurements. Data are presented in ascending order with respect to mean absolute percent error (MAPE). n, number of trials; Min, minimum; Max, maximum; IQR, interquartile range*.

Propensity for estimation bias with respect to HR is presented in Table 3, which determined significant bias for HRV4TR/ECG [−0.97 beats per min (bpm); *p* < 0.05] and CAMHRV (11.14 bpm; *p* < 0.05). The apparent underestimation from HRV4TR/ECG was <1 heartbeat away from mECG values and the 95% CI was remarkably close to zero (−1.47 to −0.47 bpm), which suggests statistical significance was observed due to low variations in reporting HR thus measurements still report at an acceptable degree of accuracy. Contrarily, poor HR data from CAMHRV (as discussed above) translated to a significant overestimation of HR (11.14 bpm) despite an especially large LOA Range (38.58 bpm). All other devices did not significantly over or underestimate HR as FSTBT (0 bpm), ELT/ECG (−0.32 bpm), ELT/PPG (0.34 bpm), and HRV4TR/PPG (−0.10 bpm) all fell within one beat per min with respect to the mECG values.

**Table 3:** Bland altman statistics: mean heart rate.

**Metric**	**Device**	***n***	**Bias (95% CI)**	**Adjusted *p*-value**	**Lower LOA (95% CI)**	**Upper LOA (95% CI)**	**LOA range**
HR	FSTBT	21	0 (0, 0)	NA	0 (0, 0)	0 (0, 0)	0
	ELT/ECG	19	−0.32 (−0.71, 0.08)	1	−1.92 (−2.57, −1.28)	1.29 (0.65, 1.93)	3.21
	HRV4TR/ECG	21	−0.97 (−1.47, −0.047)	0.008*	−3.13 (−3.95, −2.31)	1.19 (0.37, 2.01)	4.32
	ELT/PPG	21	0.34 (−0.29, 0.97)	1	−2.39 (−3.42, −1.35)	3.07 (2.03, 4.10)	5.45
	HRV4TR/PPG	20	−0.10 (−0.85, 0.65)	1	−3.23 (−4.45, −2.01)	3.04 (1.82, 4.26)	6.27
	CAMHRV	23	11.14 (6.88, 15.40)	<0.001*	−8.15 (−15.13, −1.18)	30.43 (23.45, 37.41)	38.58

The bias and lower and upper limits of agreement (LOA), along with the corresponding 95% confidence intervals (CI) for each, are included for all the devices. Instances in the “Adjusted p-value” column denoted with “^*^” symbolize statistical significance (p < 0.05), which in this case indicates significant bias (over or underestimation). The letter “n” denotes the number of trials that were examined for each device following extreme outlier detection. bpm, beats per min; 95% CI, 95% confidence interval; NA, not available; LOA, limits of agreement.

### Evaluation of COTS Devices in Reporting rMSSD

**Figure 2:**
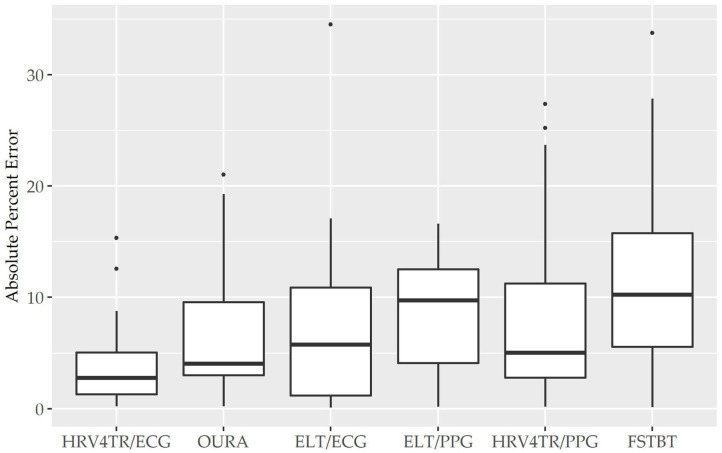
Root mean square of successive differences absolute percent error: box plot visualization of absolute percent error (APE) for the commercial-off-the-shelf (COTS) devices that reported root mean square of successive differences (rMSSD) as an indicator of heart rate variability (HRV). COTS devices that are presented include HRV4Training via ECG (HRV4TR/ECG), OURA Smart Ring (OURA), Elite HRV via electrocardiogram (ELT/ECG), Elite HRV via photoplethysmography (ELT/PPG), HRV4Training via PPG (HRV4TR/PPG), and Firstbeat (FSTBT).

To determine device capabilities for assessing rMSSD, APE values were again calculated for the six COTS devices, which are sorted in ascending order from the lowest MAPE value (HRV4TR/ECG: 4.10%) to the highest (CAMHRV: 112.36%) in Figure 2 below. Further examination of the APE summary statistics in Table 4 (APE summary table for rMSSD) revealed that HRV4TR/ECG and OURA possessed the lowest MAPE, median percentage and IQR relative to all other COTS devices (4.10, 2.76, 3.74% and 6.84, 4.06, 6.55%, respectively). Similarly, ELT/ECG (7.66%), ELT/PPG (8.71%), and HRV4TR/PPG (9.43%) all possessed MAPE values <10% whereas FSTBT was close with a MAPE of 11.27%. The inability for CAMHRV to measure HR accurately, as depicted in Figure 1 and Tables 2, 3, translated to its inability to report rMSSD measurements. MAPE for CAMHRV exceeded 100% (112.36%) while maximum error was recorded as 328.67% (after extreme outlier removal). CAMHRV was omitted from Figure 2 due to the drastically large degree of error relative to the other COTS devices such that figure scaling would have been severely limited thus hindering interpretations.

**Table 4:** Absolute percent error executive summary statistics: rMSSD.

**Metric**	**Device**	***n***	**MAPE (%)**	**Min. (%)**	**Median (%)**	**Max. (%)**	**IQR (%)**
rMSSD	HRV4TR/ECG	21	4.10	0.21	2.76	15.35	3.74
	OURA	25	6.84	0.21	4.06	21.02	6.55
	ELT/ECG	18	7.66	0.12	5.75	34.54	9.72
	ELT/PPG	22	8.71	0.15	9.73	16.63	8.44
	HRV4TR/PPG	18	9.43	0.17	5.03	27.37	8.46
	FSTBT	25	11.27	0.13	10.25	33.77	10.19
	CAMHRV	25	112.36	1.06	75.77	328.67	197.35

*Absolute percent error summary statistics are reported for all devices that provided rMSSD measurements. Data are presented in ascending order, with respect to mean absolute percent error (MAPE). n, number of trials; Min, minimum; Max, maximum; IQR, interquartile range*.

**Figure 3:**
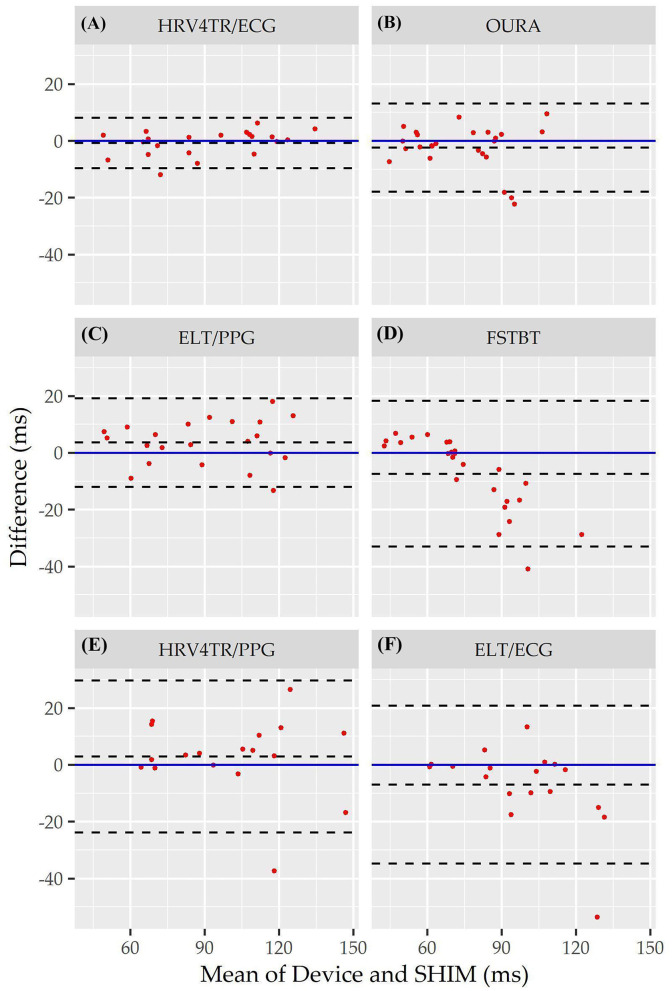
Root mean square of successive differences Bland Altman plots: visualization driven by Bland Altman analysis for the commercial-off-the-shelf (COTS) devices that reported root mean square of successive differences (rMSSD) as an indicator of heart rate variability (HRV). COTS devices that are presented include HRV4Training via ECG (**A**; HRV4TR/ECG), OURA Smart Ring (**B**; OURA), Elite HRV via photoplethysmography (**C**; ELT/PPG), Firstbeat (**D**; FSTBT), HRV4Training via PPG (**E**; HRV4TR/PPG), and Elite HRV via electrocardiogram (**F**; ELT/ECG).

**Figure 4:**
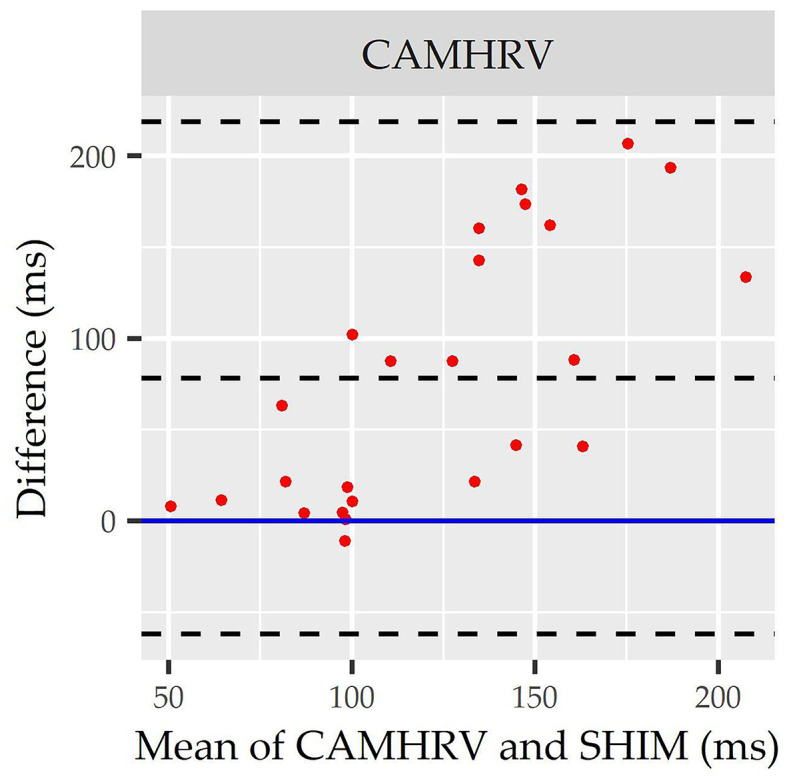
Root mean square of successive differences CameraHRV Bland Altman. Visualization driven by Bland Altman analysis for measurements of root mean square of successive differences (rMSSD) via CameraHRV (CAMHRV).

Individual Bland Altman plots for the COTS devices arranged from smallest to largest LOA range when measuring rMSSD are provided in Figures 3A–F whereas the worst performing device, CAMHRV appears separately in Figure 4 due to the drastically larger degree of error and axis scaling. Additionally, Bland Altman summary statistics providing additional insights for each COTS device are presented in Table 5.

**Table 5:** Bland altman summary statistics: rMSSD.

**Metric**	**Device**	***n***	**Bias (95% CI)**	**Adjusted *p*-value**	**Lower LOA (95% CI)**	**Upper LOA (95% CI)**	**LOA range**
rMSSD	HRV4TR/ECG	21	−0.74 (−2.79, 1.30)	1	−9.55 (−12.88, −6.21)	8.06 (4.73, 11.40)	17.61
	OURA	25	−2.33 (−5.60, 0.94)	1	−17.85 (−23.23, −12.47)	13.19 (7.82, 18.57)	31.04
	ELT/PPG	22	3.62 (0.10, 7.14)	0.58	−11.94 (−17.70, −6.19)	19.18 (13.43, 24.94)	31.13
	FSTBT	25	−7.37 (−12.77, −1.97)	0.12	−33.00 (−41.88, −24.12)	18.27 (9.39, 27.15)	51.27
	HRV4TR/PPG	18	2.96 (−3.83, 9.75)	1	−23.79 (−34.77, −12.81)	29.71 (18.73, 40.70)	53.50
	ELT/ECG	18	−6.98 (−14.02, 0.06)	0.67	−34.74 (−46.13, −23.34)	20.78 (9.38, 32.17)	55.52
	CAMHRV	25	78.33 (48.74, 107.92)	<0.001*	−62.16 (−110.84, −13.49)	218.83 (170.15, 267.50)	280.99

Tabular presentation of the bias, lower and upper limits of agreement (LOA), and their respective 95% confidence intervals (CI), per device. Instances in the “Adjusted p-value column” that are denoted with “^*^” symbolize statistical significance (p < 0.05).

Consistent with performing as the most accurate for measuring rMSSD per APE statistics, biases were the lowest for HRV4TR/ECG [Figure 3A; −0.74 milliseconds (ms)] and OURA (Figure 3B; −2.33 ms), their respective 95% confidence intervals for bias both contained zero and they had the lowest LOA ranges (Table 6). Bland Altman analyses for rMSSD also revealed that CAMHRV (Figure 4) was the only device with significant bias, which was denoted as an overestimation of rMSSD by 78.33 ms (*p* < 0.05). Overall, devices maintained consistent performance when measuring average HR and rMSSD (e.g., if average HR was accurate then rMSSD was accurate), excluding FSTBT, which was a top performer for HR (MAPE = 0%) yet faulted in performance for rMSSD (MAPE = 11.27%).

**Table 6:** Lin's Concordance Correlation Coefficient (CCC): rMSSD.

**Metric**	**Device**	***n***	**Concordance**
rMSSD	HRV4TR/ECG	21	0.98
	ELT/PPG	22	0.94
	OURA	25	0.91
	HRV4TR/PPG	18	0.87
	ELT/ECG	18	0.77
	FSTBT	25	0.76
	CAMHRV	25	0.04

*The precise CCC values for each COTS device are presented to denote the overall strength of agreement (or lack thereof) between COTS device measurements and the mECG standard device. The closer the CCC is to +1, the stronger the agreement*.

**Figure 5:**
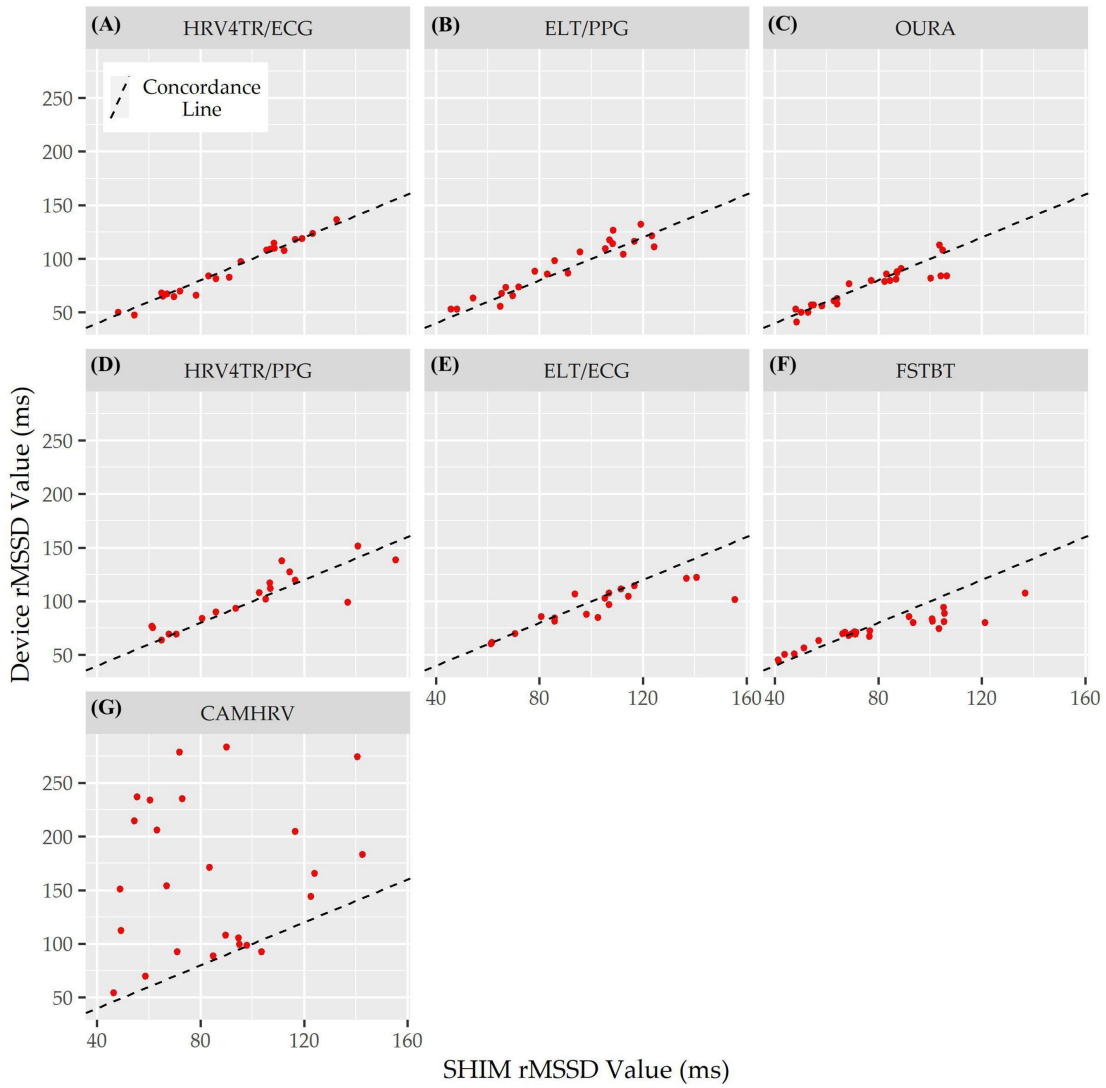
Root mean square of successive differences lin's concordance correlation coefficient. Concordance between commercial-off-the-shelf (COTS) devices and the reference standard multi-lead electrocardiogram (Shimmer; SHIM) for measurements of root mean square of successive differences (rMSSD). COTS devices include HRV4Training via ECG (**A**; HRV4TR/ECG), Elite HRV via photoplethysmography (**B**; ELT/PPG), OURA Smart Ring (**C**; OURA), HRV4Training via PPG (**D**; HRV4TR/PPG), Elite HRV via electrocardiogram (**E**; ELT/ECG), Firstbeat (**F**; FSTBT), and CameraHRV (**G**; CAMHRV).

Lastly, the Concordance Correlation Coefficient (CCC) for COTS devices that measured rMSSD provided a singular summary statistic denoting the degree of agreeance (concordance) between individual COTS devices and the mECG (Figure 5 and Table 6). Again, HRV4TR/ECG (0.98) was determined to be the strongest amongst COTS devices for reporting rMSSD, although ELT/PPG (0.94) and OURA (0.91) both performed well enough to garner CCC's >0.90. HRV4TR/PPG was the COTS device displaying the next highest CCC, which was 0.87. CCC also provided additional corroboration for the inability of CAMHRV (0.04) to measure rMSSD. ELT/ECG (0.77) and FSTBT (0.76) both performed similarly with respect to CCC, although for precise physiological measurements CCCs below 0.90 are not ideal.

## Discussion

The manufacturing and marketing of commercial off-the-shelf (COTS) wearables asserting capabilities of precise physiological monitoring via heart rate variability (HRV) typically avoids third-party evaluations of accuracy (De Arriba-Pérez et al., 2016; Henriksen et al., 2018; Bent et al., 2020). Consequently, end-users, such as the general consumer, elite performers practitioners, researchers, and clinicians that purchase COTS devices with the intent of deploying strategies to augment certain health metrics based on derived insights, are severely restricted in their knowledge of device capabilities. The present study aimed to assess the validity of various COTS devices (both tangible devices and third-party software applications) when measuring rMSSD, a common heart rate variability (HRV) metric consistently reported across the wearables market (Bent et al., 2020). Prior to examining device accuracy with respect to rMSSD, however, evaluations of mean heart rate (HR) were also conducted to provide additional evidence as to why a COTS device can or cannot successfully report rMSSD. Comparisons between cECG and PPG devices were also pertinent, provided those two types of technologies are the most commonly available across the commercial market.

COTS devices and an mECG validation standard device were deployed while study participants rested in a seated position, and later compared to ascertain which, if any, COTS devices demonstrated reasonable utility for measuring rMSSD. The primary finding in this study was that HRV4TR/ECG and OURA were consistently among the top performers when reporting rMSSD, as evidenced by the lowest mean absolute percent error (MAPE) values (See Figure 1 and Table 2), lowest limits of agreement (LOA) ranges with unremarkable biases as demonstrated by the Bland Altman analysis (See Table 3), and possessing remarkably high CCC values (Table 6). Although evaluations for mean HR from OURA were not available in the present study, it may be inferred that OURA was capable of accurate HR assessments or it is likely that rMSSD would not have been as accurate as the data indicated. Moreover, the remaining top two devices when reporting rMSSD, HRV4TR/ECG and ELT/ECG (according to MAPE), both measured mean HR with a MAPE value <5% (2.34 and 0.69%, respectively). Conversely, CAMHRV was consistently the worst performer for all of the aforementioned analyses with respect to rMSSD, which was further corroborated by a poor mean HR measurement (See Figure 2 and Tables 2, 3). For the two devices that allowed both cECG and fPPG options, the cECG based versions were able to estimate rMSSD more accurately compared to their PPG and fPPG based counterparts. However, there was no clear distinction among device type efficacy when evaluating HR.

Indeed, there are a number of technical and logistical considerations to contemplate when determining whether or not a popular COTS device is suitable for any given application(s). Please refer back to Table 1 for device pairing specifications if necessary. Notably, Elite HRV and HRV4Training were paired with a Polar H10 strap and used alone as an fPPG based device with an iPhone 8. We did not assess the efficacy of the Polar H10 strap at collecting data, rather the phone applications at their ability to process the data. The Polar H10 has already been validated by previous researchers (Gilgen-Ammann et al., 2019; Speer et al., 2020) and has been used by researchers as a standard criterion device for quantifying cardiovascular metrics (Müller et al., 2019; Weaver et al., 2019). The observed bias for CAMHRV was higher than previous research conducted on CAMHRV which reported 6 ms bias when compared to an FDA-approved pulse oximeter (Pai et al., 2018). The larger bias observed in the present study may be attributed to the previous utilization of a pulse oximeter rather than the preferred methodological comparison strategy that uses mECG. When validated against Kubios HRV 2.2, previous research reported a mean bias of −2.7% residuals when pairing Elite HRV phone application with the Polar H7 c-ECG device (Perrotta et al., 2017), which offers consistencies with our findings. To our knowledge, no validation studies have been conducted with Elite HRV using their fPPG based technology.

Findings in the present study deviated from previous research in which the FSTBT textile strap was strongly correlated to a 3-lead mECG (0.99 Pearson correlation coefficient) for both HR and rMSSD. That study also provided a Bland Altman plot of rMSSD illustrating that all but one value was observed within the mean ± 1.96 SD threshold, supplemented with tighter CI bounds (Bogdány et al., 2016) compared to the findings in the present study. Previous studies validated HRV4Training with a clinical grade mECG device and reported similar trends to those found here. Namely, the mean bias was previously reported as −1.5 and 1.4 ms when paired with a Polar H7 cECG and a smartphone camera (fPPG), respectively, while participants breathed normally (Plews et al., 2017). In both cases, HRV4Training underestimated rMSSD when paired with a cECG device compared to when it was used as a fPPG device and subsequently overestimated rMSSD. The Bland Altman analysis for OURA indicated a narrow 95% confidence interval (−5.6, 0.94 ms) with negligible bias (−2.33 ms), which supports previous research that reported a 95% confidence interval spanning from −8.8 to 6.5 ms when compared to mECG (Kinnunen et al., 2020).

Consistent with APE and Bland Altman analysis, CCC was also determined to be markedly high for HRV4TR/ECG as well as OURA. Findings in this present study support the extant literature that unveils inconsistencies in performance among COTS, as discussed. Due to the popularity of cECG and PPG devices, many types of technologies were included in the direct comparisons to mECG. Considering our study design, there are factors to recognize with respect to the different devices, their hardware and software technicalities, and their standard procedures for HRV (rMSSD) data collection. Namely, there are inherent differences between cECG and PPG COTS wearables and variations in duration of signal acquisition.

It is crucial to realize the fundamental differences between cECG- and PPG-based technologies. In addition to the hardware differences of COTS devices, of equal importance are the shape and tendencies of cECG and PPG physiological signals. ECG waveforms are denotated with a characteristic peak, whereas PPG waveforms are sinusoidal by nature. This distinction has profound implications on the signal processing capabilities that must be utilized to distinguish subsequent peaks from one another. PPG based devices require more robust processes that compensate for the lack of distinction from one heartbeat to the next (Kher, 2019). Both cECG and PPG COTS wearables are subject to signal disruption, though ECG is generally associated with a better signal to noise ratio (SNR) (Kher, 2019). ECG signals can be corrupted with baseline drift induced by chest movements during respiration, electrode contact movement, and increased electrode impedance (Allen, 2007) whereas PPG COTS are known to be especially susceptible to motion artifact, ambient light, and skin complexions (Lee and Zhang, 2003; Castaneda et al., 2018; Bent et al., 2020). Provided the known challenges with motion artifact in PPG COTS devices, our study analyzed subjects at rest thus establishing ideal environmental conditions for evaluating the capabilities of cECG and PPG COTS devices in estimating rMSSD and HR. Our data demonstrates that cECG based devices performed more accurately than their fPPG counterparts (cases where the COTS devices used the same application, like Elite HRV and HRV4Training). FSTBT and OURA were exceptions to the observed trends in cECG and PPG performance for measuring rMSSD. More specifically, OURA elicited the lowest MAPE for rMSSD among all PPGs and demonstrated a high degree of concordance with the mECG standard (See Table 6). The Oura smart ring uses infrared optical sensors (~900 nm wavelength), which penetrate the skin more deeply (Kinnunen et al., 2020) than conventional PPG devices using green LED (~520–530 nm) (Fallow et al., 2013; Bent et al., 2020). The fPPG devices examined in this study utilized the LED from the iPhone's camera flash which is ~512 nm (McCracken et al., 2017) thus being more highly absorbed in the tissue compared to the near infrared range contained in Oura smart rings. FSTBT was an anomaly as it performed immaculately when measuring HR but failed to accurately report rMSSD. Unlike all other cECG COTS devices, FSTBT unveiled especially diminished accuracy with respect to rMSSD as it was one of the worst performers in this study (See Table 4). It is postulated that this inconsistency in performance is related to the acquisition duration of the physiological signal.

The standard short (ST) epoch to conduct HRV analysis is 5 min, as noted in the most recent recommended guidelines on optimal conditions for HRV measurement state (Shaffer and Ginsberg, 2017). This 5-min representation of HRV is used to make inferences about the SNS and PNS in relation to ANS balance while the subject is at rest (Bourdillon et al., 2017; Plews et al., 2017; Shaffer and Ginsberg, 2017). Although previous research deemed that 10-s and 1-min epoch windows were suitable for rMSSD analysis (Esco and Flatt, 2014; Pereira et al., 2016; Shaffer and Ginsberg, 2017; Georgiou et al., 2018), it is still presumed that these shorter time windows are not as accurate as the ST interval (Mayya et al., 2015). The duration of data collection varied among COTS devices and applications based on the recording period determined via company guidelines. When assessing rMSSD, FSBT and CAMHRV recorded for 3-min epochs, which indeed translated to poorer performance compared to HRV4TR/ECG, OURA, and ELT/PPG, all of which used 5-min epochs. Performance was reflected in higher MAPE, large bias, wider LOA ranges, and weaker CCCs. It appears that the duration of signal acquisition had a greater impact on device accuracy than type of technology (e.g., cECG vs. PPG). Markedly, FSTBT was the leading performer when measuring HR but then, conversely, suffered from more inaccurate measures of rMSSD. This disparity in performance may be explained by the Quick Recovery Test (commonly referred to as the “QRT”) that FSTBT utilizes for rMSSD measurements which only captured data for 3 min. For these reasons, those commercial companies that are interested in effectively assessing HRV (rMSSD in most cases) are recommended to incorporate longer duration periods (i.e., ≥5 min). The incorporation of data collection segments of at least 5 min ensures that daily decisions (e.g., sleep alterations, alternative recovery interventions, training volumes, and intensities) based upon HRV “status” are done so with a greater degree of confidence (Mayya et al., 2015; De Arriba-Pérez et al., 2016). In the present study, devices, such as FSTBT and CAMHRV, that recorded for <5 min would have likely reported more favorable MAPE and CCC values as well as smaller biases and LOA ranges.

### Future

The COTS devices in the present study were selected based on input from collaborative research partners in the field of human performance, which suggested that these devices were among the most commonly used. The authors acknowledge that there are other (and even newer versions of the same) COTS devices on the commercial market that were not included in this analysis. End-users purchasing the various COTS devices should be better informed on the accuracy of device claims (with respect to physiological monitoring). However, as mentioned before, this particular market expands at a pace that scientific evaluations struggle to keep up with. For these reasons, routine evaluations of COTS devices occurring as frequently as possible with laboratory resources devoted to maintaining and updating device libraries and inventories are crucial to the dissemination of the relevant information on device accuracy to the end-users.

Like most research, this study was conducted with inherent limitations. The subject population comprised five young and healthy adults between the ages of 20 ± 1.58 years, and of varying ethnicities. Previous research determined that variations in skin complexions and textures influence the quality of data acquisition from COTS devices. Individuals with darker skin tones are often associated with higher error rates from PPG that utilizes a green LED (Fallow et al., 2013; Bent et al., 2020). Elderly patients with fragile skin and tremors are also known to elicit bad signal recording quality (De Arriba-Pérez et al., 2016; Georgiou et al., 2018). We elected not to control for skin complexions as we pursued investigation of how COTS devices perform with respect to rMSSD in a small sample of general consumers. Our recruitment strategies on a collegiate campus also skewed age demographics such that our data are from particularly young adults. Future studies should aim to examine larger populations with variation in age and skin color, with repeated measures across all individuals. Moreover, since sample sizes varied slightly for each device (due to outlier removal), future research specifically aimed at identifying instances of COTS device vulnerability via questionable data recordings is warranted.

This study sought to analyze and compare the processed output of values from COTS devices as they are observed and interpreted via user interface by the end-user. Granted, the present study did not compare the raw data outputs used to generate the summary values of HR and rMSSD. Future research should incorporate raw signal recordings in comparison to a standardized reference device and take a deeper dive into the metrics that modulate signal quality, such as the sampling frequency, sensor type, and sensor placement. Sampling frequency, or the rate of signal obtention, modulates the resolution and subsequent quality of the data and the COTS devices examined in this study ranged from 1 to 1,000 Hz. However, the sampling frequency is not always disseminated to the end-user (as was the case in several instances herein). Alternatively, end-users must accept the sampling frequency for what it is or contact the manufacturer of the company. Sensor placement varied across COTS devices, either placed on the chest, wrist, or finger. Location of the sensor can impact the quality of the signal and its susceptibility to motion artifact. Future studies should address these variations and their subsequent impacts on accurately reporting HR and HRV metrics.

Although the preferred method of time stamping comprises instantaneous stamps triggered by a central software that would synchronize all COTS devices and the mECG, the present study was limited to manual time stamping. Additionally, we did not assess the algorithms embedded within the COTS devices and their respective signal processing capabilities, primarily due to the black-box phenomenon. Due to the proprietary nature of COTS devices, there remains a concerningly large gap in research by independent third parties that thoroughly authenticate the algorithms and signal processing techniques deployed by COTS manufacturers. Manufacturers should be encouraged to place greater emphasis on validation by independent researchers, prior to marketing unvalidated (peer-reviewed) claims and releases. Researchers and manufacturers alike must participate in more symbiotic collaborations, to benefit the end-users of the COTS devices, through engaging in independent research that examines data acquisition and processing methodologies. The present study was limited in that it merely compared summary values derived from COTS devices to mECG. Ideally, synchronized (chronologically) raw data streams from the mECG and the COTS devices are compared. When accurately and objectively obtained, wearable technologies have the potential to provide non-invasive, continuous, real-time monitoring. The quantification of daily stress and recovery balance in mass general populations affords potentially valuable data to infer health or performance outcomes and drive decision-making.

Lastly, the present study specifically emphasized rMSSD from COTS devices. However, there still remains a litany of other HRV metrics, such as high frequency (HF) power, low frequency (LF) power, total power (TP), and HF/TP to be assessed in addition to continually evaluating rMSSD. The aforementioned metrics can provide additional insight into the autonomic regulation of the body beyond analyzing rMSSD alone. Despite the more robust HRV knowledge delivered by the accurate reporting of these various metrics, most COTS devices significantly limit what values are reported to the end-user. This is also not accounting for the notion that often times COTS devices simply display a value referred to as “HRV” without any further context (i.e., is it rMSSD or SDNN or some other HRV metric?) thus forcing the end-user to find out for themselves. While the common HRV metric, rMSSD, was the main focus of this study, future investigations of COTS devices that extend their data reporting beyond rMSSD are warranted.

## Conclusions

As the wearable and health industry continues to expand, end-users are allocating considerable amounts of resources (e.g., time, finances) to stress related data tracking. Examples include daily and/or nightly HRV assessments to ascertain individual levels of readiness and fatigue as well as average heart rates during sleep (Shaffer and Ginsberg, 2017; Bent et al., 2020). These models have the capability to provide objective, actionable insight to personalize stress mitigation strategies through accurate obtention and analysis of HRV and HR related metrics. Additionally, these metrics provide significant insight into performance and recovery ramifications across athletic and elite performing populations that are used in training and workload outcomes. The consumer market for COTS wearables is likely to continue its unprecedented rate of expansion; thus, it is imperative that independent, third-party evaluations for device accuracy are relentless and expeditious in their efforts. Our study aimed to assess the accuracy of numerous COTS devices and applications when reporting rMSSD and HR, compared to mECG. Similar to previous studies, devices performed with varying degrees of veracity with MAPE ranging from 4 to 112%. The greatest degrees of confidence are extended to HRV4TR/ECG and OURA, as our data suggests they can most accurately report rMSSD as both possessed MAPEs below 7% and CCCs above 0.90. Contrarily, CAMHRV was the worst performer with a MAPE of 112% and CCC of 0.04%. Further, cECG based devices generally outperformed PPGs, although there were a couple of exceptions. The Oura smart ring (PPG) exhibited better accuracy than all cECGs except for HRV4TR/ECG and the FSTBT (cECG) lacked in rMSSD accuracy.

The COTS devices evaluated herein are presently retrievable from the commercial market by general consumers, researchers, practitioners, and clinicians. Due to the aforementioned varying degrees in accuracy across these devices, we recommend steadfast scientific efforts to routinely evaluate them as they are released for purchase. Assessments of accuracy similar to the one presented here provide critical information to the end-users such that they are able to align their expectations with the inherent limitations of COTS capabilities. Device purchasing comes down to individual decision making, which is most effectively executed and subsequently incorporated into daily living, clinical monitoring, sports/military training, etc. when those persons are presented with thorough and applicable analysis. Ultimately, a decision is to be made as to what degree of accuracy is necessary for the intent of the device, which may vary across populations.

## Data Availability Statement

The raw data supporting the conclusions of this article will be made available by the authors, without undue reservation.

## Ethics Statement

The studies involving human participants were reviewed and approved by West Virginia University Institutional Review Board. The patients/participants provided their written informed consent to participate in this study.

## Author Contributions

JS, HU, AT, MH, MS, VF, SG, AR, and JH: conceptualization. JS, KT, AT, and JR: data curation. JS, HU, AT, JR, SG, and JH: formal analysis. VF, SG, AR, and JH: funding acquisition. JS, HU, KT, AT, SG, and JH: investigation. JS, KT, AT, VF, SG, and JH: methodology. JS, VF, SG, AR, and JH: project administration. JS, SG, and JH: resources. JS and KT: software. JS, AT, VF, SG, and JH: supervision. JS and KT: validation. JS, JR, and JH: visualization. JS, HU, KT, AT, MH, JR, MS, VF, SG, AR, and JH: writing – original draft. JS, HU, AT, MH, JR, MS, and JH: writing – review and editing. All authors contributed to the article and approved the submitted version.

## Conflict of Interest

The authors declare that the research was conducted in the absence of any commercial or financial relationships that could be construed as a potential conflict of interest.
